# Prevalence, Species Diversity, and Molecular Characterization of Viruses Infecting Phlox (*Phlox paniculata*) from Botanical Gardens in Russia

**DOI:** 10.3390/plants15172550

**Published:** 2026-08-22

**Authors:** Elena Motsar, Anna Sheveleva, Fedor Sharko, Svetlana Tsygankova, Irina Mitrofanova, Sergei Chirkov

**Affiliations:** 1 Virology Department, Lomonosov Moscow State University, Moscow 119234, Russia; elena.motsar31@gmail.com (E.M.); anncsh@yandex.ru (A.S.); 2 National Research Center “Kurchatov Institute”, Moscow 123182, Russia; fedosic@gmail.com (F.S.); svetlana.tsygankova@gmail.com (S.T.); 3 Tsitsin Main Botanical Garden of Russian Academy of Sciences, Moscow 127276, Russia; irimitrofanova@yandex.ru

**Keywords:** floriculture, phlox, plant virome, alfalfa mosaic virus, Arabis mosaic virus, beet ringspot virus, cucumber mosaic virus, tobacco streak virus, tobacco rattle virus, high-throughput sequencing

## Abstract

Phlox (*Phlox* spp., *Polemoniaceae*) are herbaceous ornamental plants that are cultivated around the world for their fascinating flowering. *P. paniculata* cultivar collections at the Tsitsin Main Botanical Garden and the Botanical Garden of Lomonosov Moscow State University (both in Moscow, Russia) were surveyed for virus diseases using high-throughput sequencing and RT-PCR. Twenty-seven plants with virus-like symptoms on the leaves were selected for analysis. Alfalfa mosaic virus (AMV), Arabis mosaic virus (ArMV), ArMV satellite RNA (satRNA), beet ringspot nepovirus (BRSV), BRSV satRNA, cucumber mosaic virus (CMV), Spiranthes mosaic virus 3 (SpiMV3), tobacco streak virus (TSV), and tobacco rattle virus (TRV) were identified. AMV and TSV were the most common viruses found in 22 and 25 out of the 27 samples tested, respectively. ArMV satRNA was detected in phlox for the first time, adding to the list of viruses infecting this crop. The nearly complete genomes of the detected viruses and satRNAs were assembled and characterized. The CMV and TRV genome sequences from phlox were first deposited in GenBank. Only RNA1 was detected in the TRV-infected ‘Fligelleutenant Bolke’ cultivar plant, suggesting that the so-called non-multiplying infection has been found for the first time in a plant species other than solanaceae.

## 1. Introduction

Phlox (genus *Phlox*, family *Polemoniaceae*) is a popular herbaceous ornamental crop. Native of North America, phlox is now cultivated worldwide for landscape and garden design, as well as for cut flowers and potted plants, owing to its vivid colors, abundant and long-lasting flowering and adaptability to various growing conditions. The flower’s color range varies from pure white to deep purple, crimson or violet shades. From approximately 70 known species, the annual *P. drummondii* Hook., as well as the perennial *P. paniculata* L. and *P. subulata* L. are considered the principal ones, each of which is represented with numerous cultivars [1,2].

Virus diseases of ornamental plants can reduce their productivity, esthetic appeal, and marketability. Moreover, infected ornamentals can be a natural virus reservoir that pose a threat to susceptible surrounding plants. Sap-transmissible and vector-borne viruses are of particular concern [3].

Phlox can be infected by many viruses from different taxonomic groups [4,5,6]. These include phlox S, B, and M carlaviruses, Ligustrum necrotic ringspot virus, and tobamovirus [5]; alfalfa mosaic virus [6,7,8]; tobacco rattle virus [9,10]; cucumber mosaic virus [10]; Arabis mosaic, raspberry ringspot, tobacco ringspot, and tomato ringspot nepoviruses [5,11]; Spiranthes mosaic virus 3 and Bidens mottle potyviruses [12,13]; strawberry latent ringspot virus [14]; Alternanthera mosaic virus [15]; Angelonia flower break virus [16]; tomato spotted wilt virus [17]; and Phlox pilosa virus [18]. Hydrangea ringspot virus and bean common mosaic virus genome sequences from phlox were also deposited in GenBank under accession numbers OL584367 and MT741491−MT741492, respectively (unpublished).

Furthermore, tobacco streak ilarvirus (TSV), beet ringspot nepovirus (BRSV) and BRSV satellite RNAs (satRNAs) were recently detected on phlox for the first time when viromes from symptomatic *P. paniculata* plants were analyzed using high-throughput sequencing (HTS) [19]. The nearly complete genomes of several isolates of these viruses were assembled and characterized. In addition, reads related to some other known phlox viruses were also generated in that study. Those included alfalfa mosaic virus (AMV) and cucumber mosaic virus (CMV) (genera *Alfamovirus* and *Cucumovirus*, respectively, from the family *Bromoviridae*), Arabis mosaic virus (ArMV, *Nepovirus*, *Secoviridae*), Spiranthes mosaic virus 3 (SpiMV3, *Potyvirus*, *Potyviridae*), and tobacco rattle virus (TRV, *Tobravirus*, *Virgaviridae*).

Typical of the *Bromoviridae* family, AMV, CMV and TSV have a segmented genome consisting of three single-stranded, positive-sense RNAs, each packed into separate particles. The 5′ end of genomic RNAs is capped, and the 3′ terminal sequence is organized into a tRNA-like structure. Four open reading frames (ORFs) encode replicase (ORF1), which includes methyltransferase (MTR) and helicase (HEL) motifs, RNA-dependent RNA polymerase (RdRp, ORF2a), movement protein (MP, ORF3a), and coat protein (CP, ORF3b). RNA2 of CMV and TSV also contains 3′ terminal ORF2b, which encodes the 2b protein involved in suppression of RNA silencing and long-distance transport of the viruses. These three viruses are among the most common in the world, and each has a very wide host range, including many economically important crops. In the field, AMV and CMV are transmitted by aphids, while TSV is spread through seeds, pollen, and thrips [20,21,22].

ArMV and BRSV belong to the genus *Nepovirus*, subgroups A and B, respectively [23,24,25]. Their genomes consist of two positive-sense single-stranded RNA molecules. Each RNA is covalently bound to a viral genome-linked protein (VPg) at the 5′ end, has a polyA tail at the 3′ end and encodes a polyprotein that is processed by the viral proteinase (Pro) to yield mature proteins. The RNA1-encoded polyprotein P1 is cleaved into the replicase protein containing the HEL and RdRp motifs, as well as VPg and Pro. Both ArMV- and BRSV-specific P1 polyproteins also contain two additional N-proximal domains upstream of the HEL domain, referred to as X1 and X2. The RNA2-encoded polyprotein P2 is cleaved into the CP, MP and the 5′ proximal 2A protein involved into RNA2 replication. ArMV and BRSV are soil-borne viruses that are non-persistently transmitted by root-feeding nematodes from the genera *Xiphinema* and *Longidorus*, respectively. They have a broad natural host range, ranging from annual herbaceous species to perennial woody ones [23,24].

Two types of small RNA molecules called satellite RNAs (satRNA) are often associated with nepovirus infections. Linear type B satRNAs are 1.1–1.5 kb in length and are encapsidated in virus particles. Similar to helper virus genomic RNAs, they are linked to a VPg at the 5′ end, have a poly(A) tail at the 3′ end and encode a non-structural protein of 36K–48K that is essential for satRNA replication in cooperation with the helper nepovirus replicase. Circular type D satRNAs are less than 500 nt long and encode no protein [23].

The bipartite TRV genome is represented by two positive-sense, single-stranded RNAs, the 5′ ends of which are capped, and the 3′ ends are folded into a tRNA-like structure. RNA1 of 6.8 kb long encodes replicase proteins containing MTR, HEL, and RdRp motifs, the MP and the 16K protein that is a silencing suppressor. RdRp is expressed via the ORF1 stop codon readthrough. Shorter RNA2 contains 5′ proximal ORF encoding the CP, and two downstream ORFs, which encode proteins 2b and 2c involved in the virus transmission by nematodes. However, ORFs 2b and 2c may be deleted, so the RNA2 length differs widely from 1.8 to 4.2 kb depending on the size of the retained genome segment. ORF2b, ORF2c, and the 3′ UTR can also be replaced with a 3′ portion of RNA1 due to recombination between both genomic RNAs [26]. In addition, RNA2 may be completely absent in infected plants. RNA1 encodes proteins required for virus replication and movement, thus supplying systemic virus spreading throughout the plant. In this case, viral particles are not produced, due to a lack of the CP encoded by RNA2, and the virus cannot be detected by ELISA or transmitted by nematodes. Missing RNA2 results in the so-called non-multiplying (NM) infection, which was discovered a long time ago [27] and has only been described in potatoes until now [26]. TRV has an enormous host range and is transmitted in the field by soil-inhabiting nematodes from the genera *Trichodorus* and *Paratrichodorus*.

SpiMV3 is a member of the genus *Potyvirus* in the family *Potyviridae*. The single-stranded positive-sense genomic RNA has a viral protein (VPg) covalently attached to the 5′ end and a poly (A) tail at the 3′ end. The large ORF is translated into a polyprotein, which is processed by virus-encoded proteases into ten mature proteins (P1, HCPro, P3, 6K1, CI, 6K2, VPg, NIa, NIb, and CP). Another short overlapping ORF, called PIPO, is expressed as a fused P3N-PIPO protein. Potyviruses are transmitted by aphids in a non-persistent manner [28].

The objectives of this study were to sequence and characterize the complete genomes of novel AMV, CMV, TRV, ArMV, TSV and BRSV isolates from phlox plants, as well as to survey the prevalence of these viruses in phlox collections in Russia.

## 2. Results

### 2.1. Identification of Viruses from Symptomatic Phlox Plants

**Figure 1:**
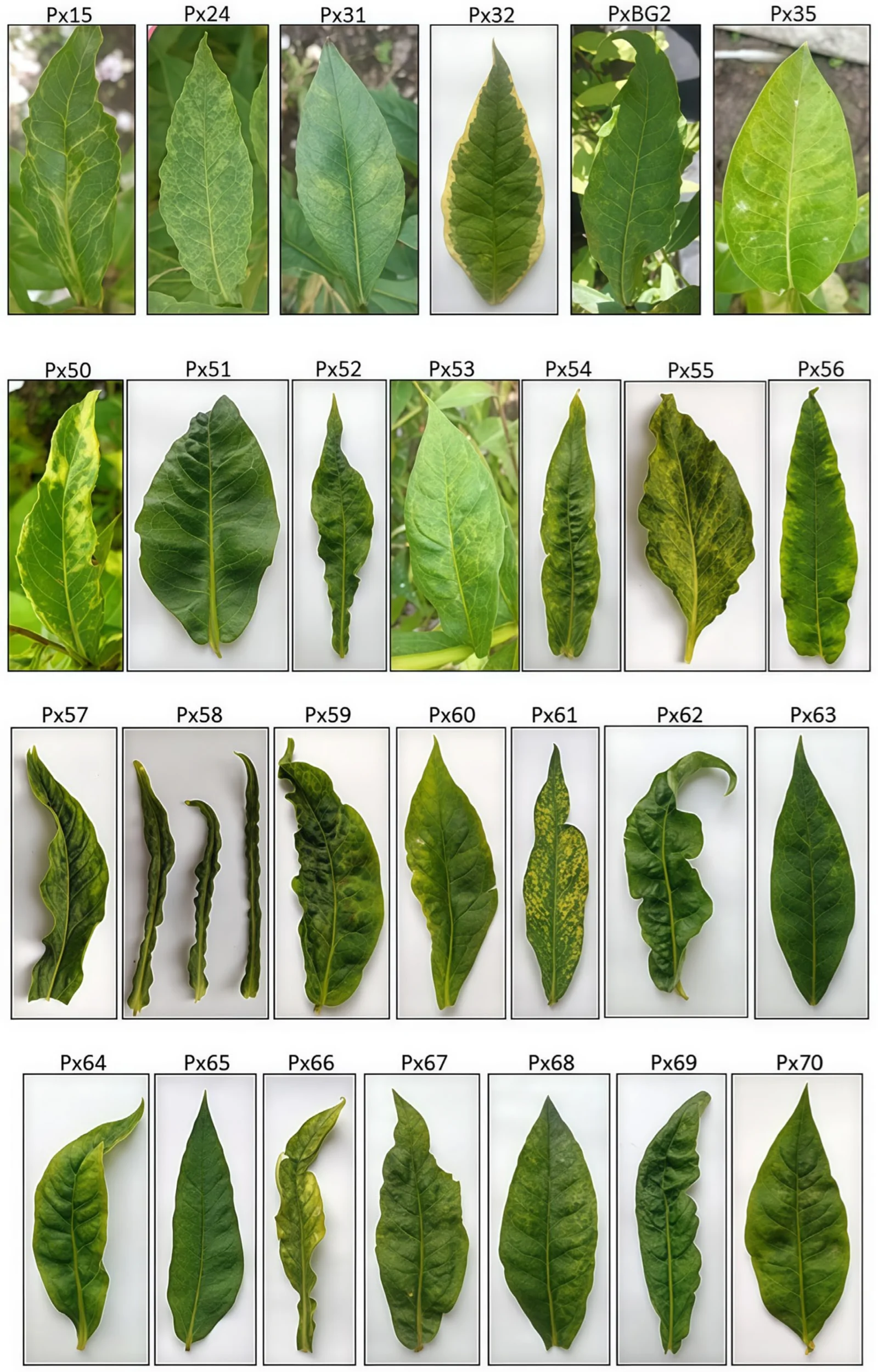
Virus-like symptoms on the leaves of *Phlox paniculata* plants. Sample names are indicated above the leaf blade photographs.

Plants with virus-like foliar symptoms, such as mottling, mosaic, rugosity, malformation, vein banding, shoestring, and chlorotic ringspots, as well as various combinations thereof, were found in phlox cultivar collections (Figure 1). Twenty-seven specimens of symptomatic leaves were collected from different cultivars for further analysis. A sample to be analysed was a single leaf with clear symptoms taken from one plant of a specific cultivar.

The viromes of six selected samples were analyzed using high-throughput total RNA sequencing (RNA-Seq). Three more samples (Px15, Px31, PxBG2) were sequenced and characterized earlier [19]. In each sample, 9–51 million raw pair-ended reads of 150 bp in length were generated, from which 11–61 thousand contigs were assembled (Table S1). The only exception was sample Px32, from which approximately 100 thousand reads were obtained, resulting in only 325 contigs. Contigs related to seven viruses (AMV, TSV, BRSV, CMV, TRV, ArMV, and SpiMV3), as well as BRSV and ArMV satRNAs, were assembled from HTS data. The raw reads were deposited in NCBI Sequence Read Archive under accession numbers SRR32918043–SRR32918045, SRR39295637–SRR39295641 and SRR39299527.

Based on these findings, each of the viruses and satellite RNAs revealed by HTS was then tested in all 27 samples using RT-PCR with virus-specific primers (Table S2). The results of the virus detection are illustrated in Figure S1. RT-PCR was performed on the template of total RNA extracted from a single leaf taken from a plant of a particular cultivar. The combined HTS and RT-PCR results are presented in Table 1. They showed that AMV and TSV were the most prevalent viruses, detected in 22 and 25 out of the 27 samples tested, respectively. SpiMV3 and BRSV were found in nine samples. BRSV satRNA and CMV were detected in five samples, TRV was found in two samples, and ArMV and ArMV satRNA were revealed in one of the samples tested. All viruses detected by HTS were confirmed using RT-PCR with virus-specific primers followed by Sanger sequencing. As a mixed infection was observed in most samples, it was impossible to attribute the foliar symptoms to the detected viruses.

Using the obtained contigs, the complete and partial genomes of the detected viruses were assembled and characterized.

### 2.2. Molecular Characterization of Detected Viruses

#### 2.2.1. Tobacco Streak Virus, Beet Ringspot Virus, and Beet Ringspot Virus Satellite RNA

TSV, BRSV and BRSV satRNA were recently found on phlox for the first time [19]. In this study, their prevalence and genetic variability in phlox cultivar collections were further investigated using HTS and RT-PCR.

TSV was the most common virus in the tested plants (Table 1). It was detected in 92.6% of the samples using RT-PCR, showing the highest incidence in the surveyed collections (Figure S1B). Three novel complete TSV genomes were assembled from the reads generated in the Px58, Px62, and Px68 samples. These were 99.8–100% identical to each other and to previously sequenced genomic RNAs of the Px15, Px31, and PxBG2 isolates [19] and had the same genome organization. In the Px32 sample, two TSV-related contigs of 2060 nucleotides (nt) in total length covered RNA3 of the closest TSV-Px15 isolate (PV524997) with 99.9% identity, encompassing both MP- and CP-encoding ORFs. The TSV genomic sequences assembled in this study were deposited in GenBank under accession numbers PZ470505–PZ470508 and PZ470511–PZ470516.

BRSV was revealed in one third of the samples tested (Figure S1C; Table 1). In addition to the BRSV genomes sequenced previously [19], another one was assembled from the Px62 sample in this study. RNA1 and RNA2 of the BRSV-Px62 isolate shared 85.0–90.7% and 89.4–91.2% identities, respectively, with other Russian BRSV isolates from phlox confirming the low level of identity between them. In the Px32 sample, two BRSV-related contigs of 539 nt and 537 nt were 89.6% to 89.9% identical, respectively, to RNA1 and RNA2 of other phlox BRSV isolates. Thus, in contrast to TSV, the Russian BRSV isolates from phlox were only distantly related to one another, suggesting either multiple independent introductions or long-term circulation of the virus within the cultivar collections, or both. The high divergence of the BRSV isolates from phlox corresponds to the global genetic diversity of this virus. The variability in the BRSV complete genome sequences deposited in GenBank ranges from 82.8% to 99.6% for RNA1 and 81.3% to 98.8% for RNA2. The assembled BRSV genomic sequences were deposited in GenBank under accession numbers PZ470517–PZ470520.

BRSV satRNA was detected in six out of nine samples infected with BRSV using RT-PCR with the satRNA-specific stBRSV-F/stBRSV-R primers (Table 1; Figure S1D), indicating that the helper virus is not always associated with the presence of satRNAs. The nearly complete BRSV satRNA sequence from the Px62 sample was recovered. It was 1373 nts long and most closely related (93% identity) to the previously characterized BRSV satRNA from the Px15 sample (PV541158) [19]. The BRSV satRNA sequence was deposited in GenBank under accession number PZ463385.

#### 2.2.2. Alfalfa Mosaic Virus

AMV was detected in 81.5% of the samples using RT-PCR with AMV coat-F/coat-R primers (Table S2), which amplified the entire CP gene (Figure S1A). The amplicons from four randomly selected samples (Px51, Px52, Px55, and Px59) of 700-bp were directly sequenced by the Sanger method. The CP gene sequences shared 98.3–99.9% nt identity.

The nearly complete AMV genomes were assembled from contigs obtained in the Px15 and Px31 samples. RNA1 of the AMV-Px15 isolate was 3601 nt long and contained ORF1 of 3381 nt encoding the 126K replicase of 1126 amino acids (aa) residues. The MTR and HEL motifs were predicted at aa positions 68–404 and 834–1094, respectively. RNA2 of 2566 nt contained ORF2 2373 nt long encoding a 90K Bromoviridae_RdRp (cd23252) of 790 aa. ORF3a and ORF3b of 903 nt and 666 nt, encoding the 32K Bromo_MP (pfam01573) and the 24K Ilar_coat CP (pfam01787) of 300 aa and 221 aa, respectively, were found in RNA3 of 2030 nt length. ORF3a and ORF3b were separated by the intergenic region of 50 nt. The genome of the AMV-Px31 isolate was nearly identical to that of the AMV-Px15. RNA1, RNA2 and RNA3 of these two isolates shared 99.4%, 99.3% and 99.5% identities, respectively, and were closest to the German PV-1411 isolate from *Origanum vulgare* (OR607775, OR607777) (RNA1, RNA3) and the Spanish L22 isolate from *Medicago sativa* (PP333070) (RNA2) with 97.2–98.8% nt identity. Despite the very wide host range, the complete genomic sequences of known AMV isolates were rather close. RNA1, RNA2 and RNA3 identities varied in ranges of 93–99%, 94–99% and 91–99%, respectively. The CP gene sequences of the AMV-Px15 and AMV-Px31 isolates were 98.8–99.8% identical to those from the Px51, Px52, Px55, and Px59 isolates, suggesting that closely related AMV isolates inhabit phlox collections. It is highly likely that the detected AMV isolates originated from a common ancestor that was once introduced into the collections and then spread between plants by aphids. Furthermore, partial AMV genomes were assembled from contigs obtained in the Px58 and Px68 samples. No substantial AMV-related contigs were assembled from the PxBG2 sample, although the virus was clearly detected by RT-PCR (Figure S1A).

The nearly complete AMV genomes from the Px15 and Px31 samples, partial genomic sequences from the Px58 and Px68 samples, and entire CP sequences from the Px51, Px52, Px55, and Px59 samples were deposited in GenBank under accession numbers PZ496784–PZ496789, PZ496794–PZ496798, and PZ496790–PZ496793, respectively. This significantly replenished the genetic information available about AMV from phlox.

**Figure 2:**
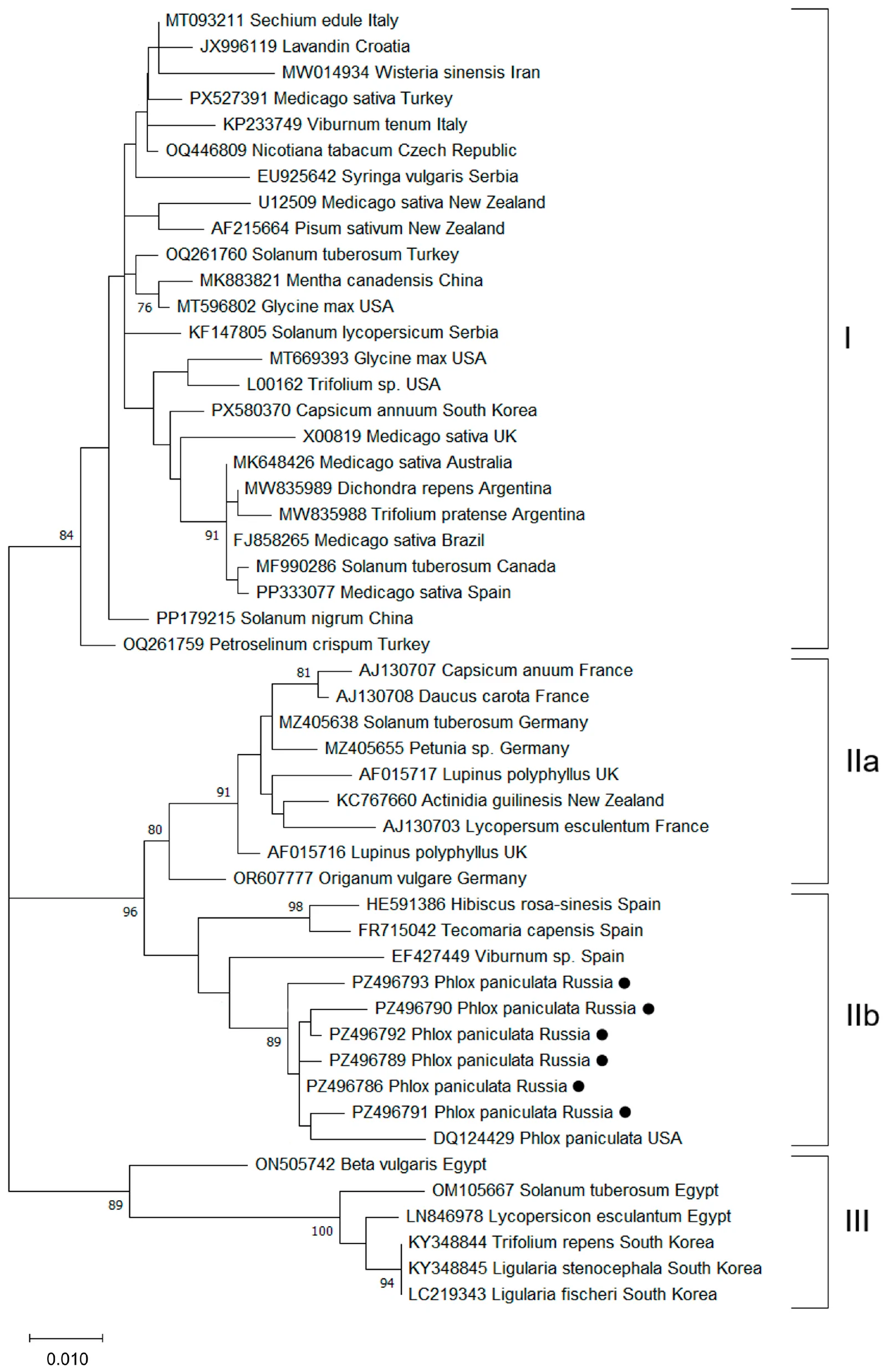
Phylogenetic analysis of alfalfa mosaic virus (AMV) isolates from phlox and selected AMV isolates from other hosts based on nucleotide sequences of the coat protein. The tree was reconstructed in MEGA X using the maximum likelihood method with the best-fit GTR substitution model. Bootstrap values (>75%) from 1000 replications are shown next to the corresponding nodes. The accession numbers in GenBank, Latin name of the host plant, and geographical origin of the virus isolate are given at the end of the branches. Brackets combine isolates of subgroups I, IIa, IIb, and III. The Russian phlox AMV isolates are marked with a black circle (•). The scale bar indicates the number of substitutions per nucleotide.

Based on phylogenetic analysis of the CP gene, the AMV isolates were shown to be clustered into groups I, IIA, and IIB according to their geographic origin [29]. Initially, group IIB was formed by Spanish isolates from Cape honeysuckle, hibiscus rosa-sinensis and viburnum. The Russian AMV isolates were assigned to group IIB, along with the American one from *P. paniculata* (DQ124429), adding to the list of hosts and geographical origin of the AMV isolates that comprise this phylogenetic group (Figure 2). Phlox isolates formed a distinct clade with high (89%) bootstrap support, suggesting host clustering of AMV isolates from *P. paniculata*. Group III is represented by relatively new AMV isolates from different hosts and geographical locations.

#### 2.2.3. Spiranthes Mosaic Virus 3

Nine Russian isolates of SpiMV3 had previously been identified in phlox and their genomes were completely or partially sequenced and characterized [13] (Table 1). No additional SpiMV3 isolates were detected in the present study by RT-PCR using primers, targeting the CP gene [30]. At the same time, a contig of 7082 nt in length spanning the genome segment from the P3 to CP genes and the 3′ UTR was assembled in the Px58 sample and deposited in GenBank under accession number PZ470509. This contig was 98.7% identical to the SpiMV3-Px24 isolate from the Fuji cultivar, whose complete genome sequence had been determined previously (PQ374234) [13]. The corresponding region of this contig was also identical to the 3′ terminal genome sequence of the SpiMV3-Px58 isolate, which was determined and deposited in GenBank under the accession number PQ464117 in our previous work [13].

#### 2.2.4. Cucumber Mosaic Virus

Five CMV isolates were found in the Px53, Px54, Px67, Px68, and Px70 samples (Table 1) using RT-PCR with TALL3/TALL5 primers [31], which amplified the entire CP gene (Figure S1E). The corresponding PCR products were sequenced. The CP gene of the detected isolates was 657 nt long and translated into a 24K protein of 218 aa. The CPs of the five isolates were highly similar, with 98.3–100% and 99.1–100% identities at the nt and aa levels, respectively.

**Figure 3:**
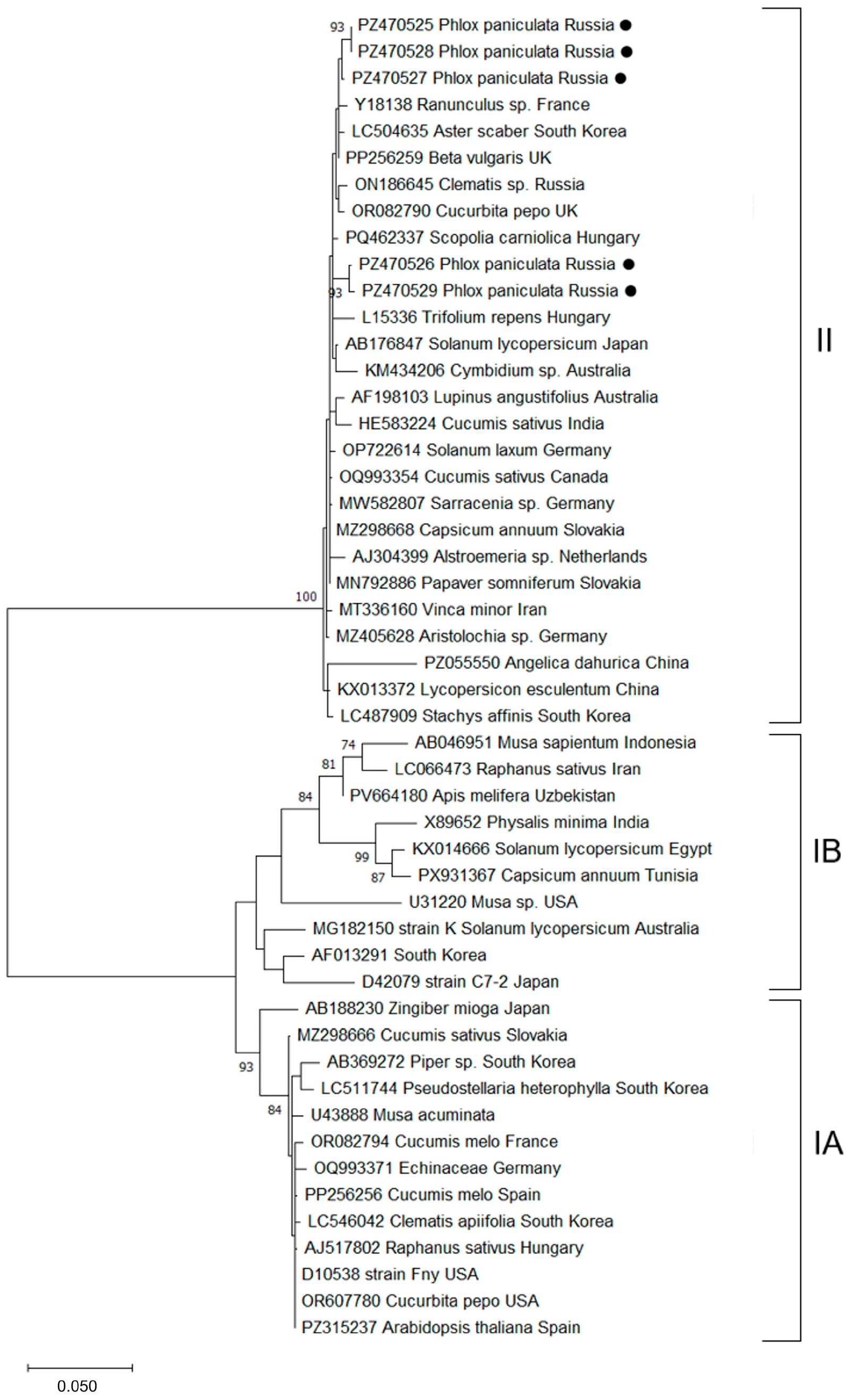
Phylogenetic analysis of cucumber mosaic virus (CMV) isolates from phlox and selected CMV isolates from other hosts based on nucleotide sequences of the coat protein. The tree was reconstructed in MEGA X using the maximum likelihood method with the best-fit GTR nucleotide substitution model. Bootstrap values (>70%) from 1000 replications are shown next to the corresponding nodes. The accession numbers in GenBank, Latin name of the host plant, and geographical origin of the virus isolate are given at the end of the branches. Brackets combine isolates of IA, IB, and II subgroups. The phlox CMV isolates are marked with a black circle (•). The scale bar indicates the number of substitutions per nucleotide.

The nearly complete CMV genome consisting of three RNAs was assembled in the Px68 sample. RNA1 of 3202 nt encoded the 111K replicase protein of 993 aa long, in which MTR (pfam01660) and HEL (pfam01443) motifs were identified. RNA2 of 2956 nt contained two ORFs. ORF2a 2574 nt long encoded the 97K Bromoviridae_RdRp (cd23252) of 857 aa. ORF2b of 333 nt overlapped the 3′ end of ORF2a and encoded a 12.7K protein 110 aa long, which contained the Cucumovirus protein 2B motif (pfam03263). RNA3 contained ORF3a and ORF3b, which code for the 30K 3A_MP (pfam00803) of 279 aa and 24K Cucumovirus_CP (pfam00760) of 218 aa, respectively. RNA1, RNA2, and RNA3 of the CMV-Px68 isolate were closest to the American pumpkin isolate (OR607778; 99% identity), French tomato isolate (HE793684; 99.3% identity), and Canadian cucumber isolate (OQ993354; 98.7% identity), respectively. Based on the CP or RNA3 phylogeny, CMV isolates were shown to be divided into subgroups IA, IB, and II [21,32]. Subgroup II is believed to be the oldest, while subgroup IB split off from it and appears to be ancestral to subgroup IA. All five Russian CMV isolates from phlox were assigned to subgroup II (Figure 3).

On the other hand, phylogenetic analysis of the nearly complete genomic RNA sequences revealed that RNA1 and RNA2 of the CMV-Px68 isolate clustered within subgroup IA, whereas RNA3 was assigned to subgroup II. This incongruency strongly suggests a reassortment event in the evolutionary history of this CMV isolate from phlox (Figure S2). Reassortment increases genetic diversity in CMV and is thought to be an important factor in its evolutionary success [33].

The complete and partial CMV genomic sequences were deposited in GenBank under accession numbers PZ470523–PZ470529. These are the first CMV genome sequences from phlox deposited in GenBank.

#### 2.2.5. Tobacco Rattle Virus

**Figure 4:**
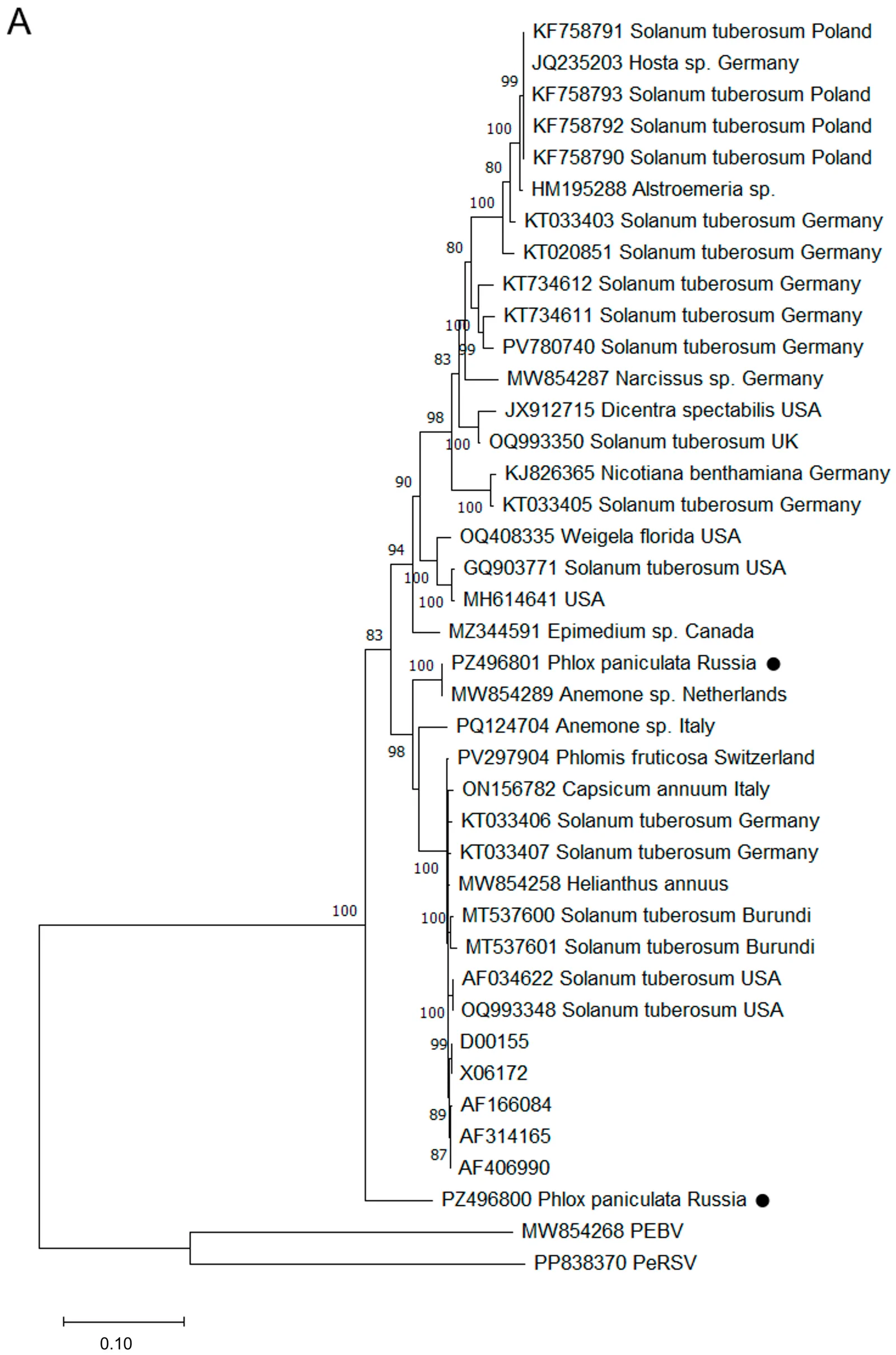

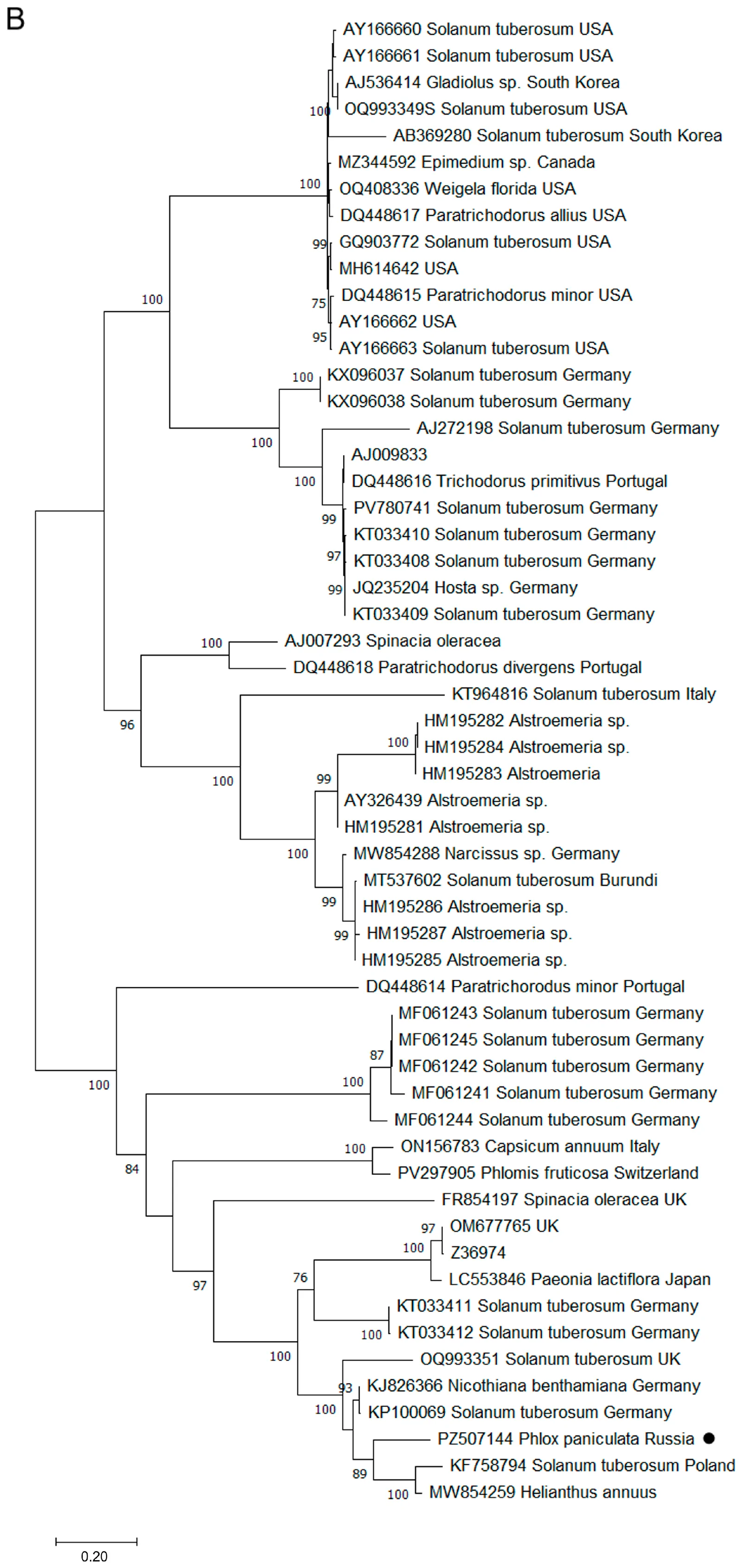
Maximum likelihood phylogenetic trees based on full-length sequences of tobacco rattle virus (TRV) genome RNA1 (**A**), and RNA2 (**B**) using the best-fit GTR nucleotide substitution model. The accession number, Latin name of the host plant, and geographical origin of the virus isolate are given at the end of the branches. TRV isolates from phlox are marked with a black circle (•). PEBV—pea early-browning tobravirus; PepRSV—pepper ringspot tobravirus. Bootstrap values from 1000 replications (>75%) are shown next to the corresponding node. The scale bar indicates the number of nucleotide substitutions per site.

TRV was detected in the Px50 and PxBG2 samples using HTS (Table 1) and confirmed by RT-PCR (Figure S1F). In the PxBG2, the TRV genome was represented by RNA1 of 6888 nt and RNA2 of 1930 nt in length. Three ORFs were predicted in RNA1. ORF1 of 3564 nt encodes the 134K replicase protein containing MTR (pfam01660) and HEL (pfam01443) domains. A readthrough translation of the ORF1 TGA stop codon at positions 3748–3750 results in the synthesis of the 194K protein containing a Virgaviridae_RdRp motif at the C-terminus. ORF2 starts one nt downstream of the 3′ end of the prolonged ORF1 and encodes the 29K MP. ORF3 encodes a cysteine-rich 16K protein, which is known as an RNA silencing suppressor. ORF2 and ORF3 are separated with an intergenic region of 24 nt. The 5′ and 3′ UTRs are 186 nt and 212 nt long, respectively. RNA1 of the TRV-Px50 isolate (6785 nt long) was organized in a similar way but was only distantly related to that of the TRV-PxBG2 sharing 91.4% identity. PxBG2 and Px50 RNAs 1 were most closely related to the Scottish potato isolate DSMZ PV-0354 (OQ993350) and to the Dutch anemone isolate DSMZ PV-1229 (MW854289), respectively, being identical to their closest relatives by 92.0% and 99.8%, respectively. Phylogenetic analysis of RNA1 sequences available in GenBank showed that the TRV-Px50 isolate clustered with the Dutch isolate from anemone, while the TRV-PxBG2 formed a separate branch (Figure 4A), although it falls within the range of variability (91–99%) characteristic of TRV RNA1 available from GenBank.

The assembled PxBG2 RNA2 sequence was verified by 3′ RACE using a forward primer targeting the 3′ end of the CP gene. RNA2 was shown to consist of the 5′ UTR 558 nt in length, ORF of 630 nt encoding the tobacco mosaic virus-like 24K CP (pfam00721), and the 3′ terminal segment of 742 nt, in which no additional ORFs were detected using the NCBI ORF finder program. Instead of this, this genomic segment (nt 1386–1886) was 100% identical to a 3′ terminal portion of RNA1 of the PxBG2 isolate (nt 6232–6732), which encompassed the 5′ sequence of a truncated 16K coding sequence and most of the RNA1 3′ UTR. The PxBG2 RNA2 was most closely related (98.9–99.3% identity) to that of the TRV isolates from potato (KF758794) and sunflower (MW854259), and formed a common clade with these isolates in phylogenetic analysis (Figure 4B). No RNA2-related contigs were assembled from the Px50 sample, and TRV was not detected using RT-PCR with CP-specific primers [33,34] (Table S2). This finding suggests that only TRV RNA1 is present in the infected plant, and the non-multiplying (NM)-like infection apparently occurs in this phlox cultivar. In laboratory conditions, TRV NM infections were also obtained in plants of the *Nicotiana* and *Petunia* genera from the *Solanaceae* family [26,27]. To the best of our knowledge, this is the first report of the TRV NM infection in a plant species other than from solanaceae. Novel TRV genomic sequences were deposited in GenBank under accession numbers PZ496800, PZ496801, and PZ507144. These are the first TRV genome sequences from phlox deposited in GenBank.

#### 2.2.6. Arabis Mosaic Virus

**Figure 5:**
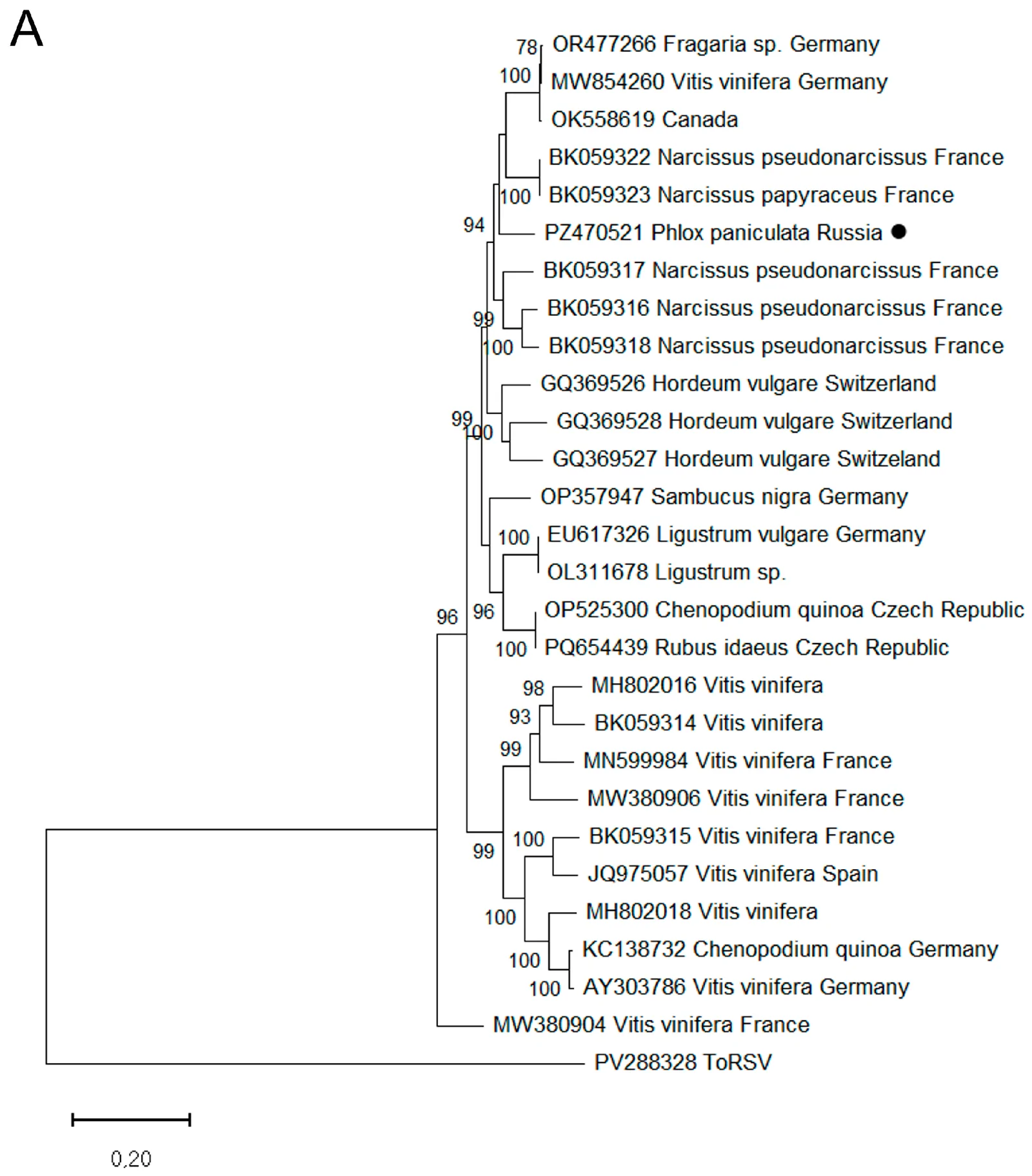

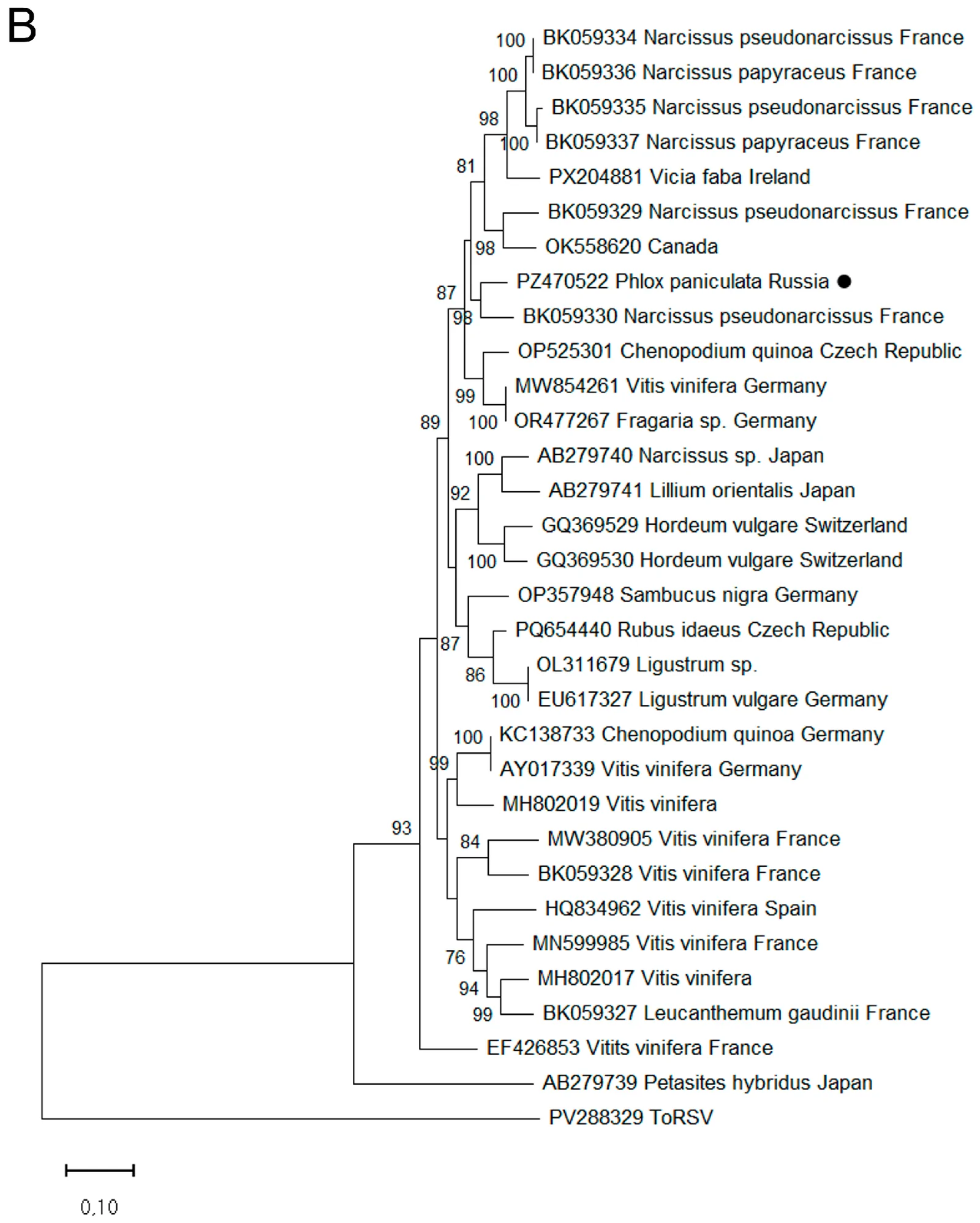
Maximum likelihood phylogenetic trees based on full-length sequences of Arabis mosaic virus (ArMV) genome RNA1 (**A**), and RNA2 (**B**) using the best-fit GTR nucleotide substitution model. The accession number, Latin name of the host plant, and geographical origin of the virus isolate are given at the end of the branches. Tomato ringspot virus (ToRSV) was used as a phylogenetical outgroup. Bootstrap values from 1000 replications (>75%) are shown next to the corresponding node. Russian phlox isolate is marked with a black circle (•). The scale bar indicates the number of nucleotide substitutions per site.

ArMV was detected in the Px24 sample (Table 1; Figure S1G). Typical of nepoviruses, the ArMV genome consisted of two RNAs 7363 (RNA1) and 3785 (RNA2) nt in length. RNA1 had a single ORF of 6852 nt, which encoded a polyprotein P1 of 2283 aa. The conserved RNA-helicase (pfam00910) and Secoviridae_RdRp (cd23196) domains were found in the P1 polyprotein at aa positions 778–881 and 1651–1958, respectively. A single ORF of 3333 nt encoding a polyprotein P2 of 1110 aa was predicted in RNA2. Nepovirus subgroup A polyprotein domain (pfam12312), nepovirus coat protein central domain (pfam03391), and nepovirus coat protein C-terminal domain (pfam03688) were identified in the polyprotein P2 at aa positions 88–259, 771–941, and 948–1106, respectively. RNA1 was most closely related (89.6% nt identity) to the isolates SRR1619673-2_ArMV from narcissus *Narcissus pseudonarcissus* (BK059317) and PV-0045 from grape *Vitis vinifera* (MW854260), although it was two consecutive triplets (nt positions 1098–1103 in ORF1) longer than its closest relatives. Similar to RNA1, RNA2 was closest (92.6% nt identity) to the French narcissus isolate SRR1619673-2_ArMV (BK059330). Despite their relatively low similarity to their closest relatives, both RNAs fall within the ranges of variability, which are 77–93% and 88–92% for RNA1 and RNA2, respectively, of known ArMV isolates. On the phylogenetic trees, RNA1 and RNA2 from the Russian ArMV-Px24 isolate were assigned to a cluster that consists mainly of French narcissus isolates (Figure 5). ArMV in the Px24 sample was confirmed by RT-PCR using RNA1- and RNA2-specific primers Ar1 and Ar2 (Table S2) designed based on the complete genome sequence of the phlox ArMV isolate (Figure S1G). ArMV genome sequences were deposited in GenBank under accession numbers PZ470521–PZ470522.

#### 2.2.7. Large Arabis Mosaic Virus Satellite RNA

Apart from ArMV, the ArMV satRNA sequence of 1101 nt long was also assembled in the Px24 sample. It was most closely related (90.3% nt identity) to an ArMV satRNA from lilac *Syringa vulgaris* (D00664) [35]. Single ORF of 1086 nt encoding a 39K protein of 361 aa was identified in the ArMV satRNA. This ORF was one triplet longer than that from lilac satRNA. The AUG start codon for this ORF was predicted at nt positions 1–3, suggesting that the 5′ UTR of the satRNA was apparently sequenced incompletely. The presence of ArMV satRNA in the Px24 sample was confirmed by RT-PCR (Figure S1G) using virus-specific primers stArMV-F/stArMV-R, which were designed based on the nearly complete satRNA sequence (Table S2). No ArMV satRNA was previously reported from ArMV-infected phlox [36]. To the best of our knowledge, this is the first detection of the ArMV satRNA from phlox. The ArMV satRNA sequence was deposited in GenBank under accession number PZ470510.

## 3. Discussion

Using HTS and RT-PCR with virus-specific primers, seven viruses and two nepovirus-associated satRNAs were identified in symptomatic plants from Russian botanical garden phlox collections. AMV and TSV were the most prevalent, being detected in the majority of the 27 samples tested. By contrast, ArMV and its associated satRNA were found in only a single plant. ArMV has been known on phlox since 2002 [37] and was shown to be widespread on *P. paniculata* co-infected with CMV, AMV and tomato spotted wilt virus [4,11]. AMV was previously found in *P. paniculata* in Poland, Lithuania, Ukraine, and the USA [4,6,7,8]. CMV infection in *P. drummondii* and *P. subulata* plants was first reported in the USA [4,5,38]. This virus was also detected on *P. paniculata*, *P. divaricata* and *P. maculata* in Israel, France and Lithuania [10,39,40]. TRV was observed in different *Phlox* spp. plants in Holland, the USA, France, Poland, Lithuania, and Ukraine [4,6,9,10]. AMV and TRV have been shown to have the highest incidence on *P. paniculata* in botanical gardens in Lithuania, Ukraine, and Poland [6]. In this study, AMV, CMV, TRV, and ArMV isolates from phlox were first reported in Russia, extending information on the global geographical distribution of these viruses. ArMV satRNA was detected in phlox for the first time, adding to the list of viruses infecting this crop. TSV, BRSV, and BRSV satRNA have been detected from phlox only in Russia to date [19].

Botanical gardens worldwide actively exchange plant material, which is not always subjected to virus testing. Therefore, it was unsurprising that the viruses identified in this study were found in both local and introduced phlox cultivars. Once introduced, a virus can be then transmitted between plants by vectors or contacts, as well as through pollen or seeds. CMV, AMV, TSV, and SpiMV3 isolates, regardless of the *P. paniculata* cultivar, showed low intra-species variability. This suggests that the isolates from each of these viruses may have a common origin. By contrast, both TRV and BRSV isolates were distantly related to each other, and apparently, each of them entered the botanical garden through several independent introductions.

Although most of the detected viruses are well-characterized and numerous genomic sequences are available in GenBank, the genomes of their phlox isolates have remained poorly studied until now. The only CP sequence of the American AMV isolate from *P. paniculata* has been submitted in GenBank (DQ124429) [8]. Only two short ArMV genome sequences (AJ090009, KP027437) were available and no TRV or CMV genome sequences from phlox have been deposited in GenBank so far. In the present study, we have obtained a significant amount of new genetic information about viruses infecting phlox. The nearly complete genomes of several AMV, CMV, TRV, and ArMV isolates as well as ArMV satRNA from different *P. paniculata* cultivars were sequenced for the first time. In addition to the TSV and BRSV genomes that were recovered previously [19], nucleotide sequences of the other TSV and BRSV isolates detected in this study were determined. Determining full-length genome sequences of phlox viruses can contribute to understanding their place among global isolates of the same viruses from other hosts, evaluating their genetic diversity and improving molecular techniques for their detection. In particular, a number of novel primers were designed to provide the reliable detection of Russian ArMV, ArMV satRNA, and TRV isolates using RT-PCR (Table S2).

Two distinct TRV isolates, TRV-PxBG2 and TRV-Px50, were found in phlox plants of unknown cultivar and the ‘Fliegelleutenant Bolke’ cultivar, respectively (Table 1). In both plants, TRV was detected using HTS and RT-PCR with primers targeting RNA1 (Figure S1). RNAs 1 of these isolates were distantly related to each other. No RNA2 was detected in the plant of the ‘Fliegelleutenant Bolke’ cultivar. Productive TRV infection in the absence of RNA2 has been discovered in potatoes and was called non-multiplying infection [27]. In this study, we have apparently found for the first time such TRV infection in plant species other than solanaceae. However, this suggestion is based on a single specimen. More TRV-infected phlox plants should be studied to confirm this finding.

The viruses reported here each have an extremely broad host range and are distributed worldwide, exerting a major impact on floriculture and posing a potential threat to numerous other crops, both related and unrelated. They are readily transmitted between plants by insect and nematode vectors, pollen and seeds, as well as mechanically and during vegetative propagation. The findings of this study may facilitate the development of appropriate phytosanitary measures to monitor and mitigate the further spread of these pathogens.

## 4. Materials and Methods

### 4.1. Sampling and Sample Processing

The living phlox cultivar collections at the Tsitsin Main Botanical Garden (MBG) of the Russian Academy of Sciences (N55.832718; E37.598346) and the Botanical Garden (BG) of Lomonosov Moscow State University (N55.844481, E37.589794), both located in Moscow, Russia, were surveyed for virus diseases from 2023 to 2025. Leaves with virus-like symptoms were collected. Most of the samples were taken from the MBG, while the Px24 and PxBG2 were from the BG. A single leaf with clear symptoms from a single plant of a specific cultivar was used for further processing. Total RNA was isolated from fresh leaves using a RNeasy Plant Mini Kit (Qiagen, Hilden, Germany) according to the manufacturer’s instructions and stored at −70 °C until use.

### 4.2. High-Throughput Sequencing (HTS)

cDNA libraries were constructed from total RNA using a TruSeq Stranded Total RNA Library Prep Plant kit (Illumina, San Diego, CA, USA) according to the manufacturer’s protocol. Sequencing was performed on an Illumina NovaSeq 6000 (Illumina, San Diego, CA, USA) platform at the National Research Center “Kurchatov Institute” in Moscow, Russia to generate 150-nt paired-end reads. Raw reads were quality-trimmed and filtered using FastQC v.0.11.9 and Trim Galore v.0.6.5 (https://www.bioinformatics.babraham.ac.uk/projects/trim_galore; accessed on 19 January 2026) with default parameters. Clean reads were assembled de novo into contigs using metaSPAdes v.3.15 [41]. Putative virus-related contigs were identified by BLASTn (2.17.0) and BLASTx (2.17.0) searches against the NCBI non-redundant nucleotide (nt) and protein (nr) databases.

### 4.3. Reverse Transcription Polymerase Chain Reaction (RT-PCR)

Isolated total RNA was also used for the virus detection by RT-PCR. The first cDNA strand was synthesized using random hexamer primers and Moloney murine leukemia virus (MMLV) reverse transcriptase (both from Evrogen, Moscow, Russia). PCR was performed using the proofreading Encyclo DNA polymerase (Evrogen) and virus-specific primers, taken from published articles and/or developed based on nearly complete genome sequences determined in this study (Table S2). Total RNA from uninfected phlox plants was a negative control. PCR products were resolved by electrophoresis in 1.5% (*w*/*v*) agarose gel, stained with ethidium bromide and photographed using a MultiDoc-It instrument (Analytik Jena US LLC, Upland, CA, USA). PCR products of the expected sizes were purified from agarose gel using a BC022 Cleanup Standard Kit (Evrogen) and sequenced in both directions by the Sanger method using Evrogen facilities.

### 4.4. Sequencing of the *3′* Ends of Virus RNA

The 3′ terminal sequence of TRV RNA2 was determined using a SKS03 Mint RACE cDNA amplification set (Evrogen; https://evrogen.ru/products/cdna/race/mint-race-cdna) (accessed on 3 March 2026) and the forward primer 5′-GTGCGTCCTAATCCTTGATGT-3′, specific to the 3′ portion of the CP gene, according to the manufacturer’s recommendations. Prior to the 3′ RACE, the 3′ ends of RNAs were polyadenylated using M0276S *E. coli* poly(A) polymerase (New England BioLabs, Ipswich, MA, USA) following the manufacturer’s instruction.

### 4.5. Sequence Analyses

For an analysis of newly assembled virus genomes, full-length genome sequences of other isolates of these viruses were retrieved from GenBank. Multiple alignments of nucleotide sequences were carried out using the ClustalW algorithm [42]. The alignments were used to calculate nucleotide identities between viral isolates and for phylogenetic analyses conducted in MEGA X [43]. Phylogenetic trees were reconstructed using the maximum likelihood method with 1000 bootstrap replications [44]. The best-fit GTR nucleotide substitution model was selected using the Jmodeltest program [45]. ORFs in the viral genomes were identified using the NCBI ORF finder program (https://ncbi.nlm.nih.gov/orffinder, accessed on 2 February 2026). The conserved domains in viral proteins were searched using the NCBI Conserved Domain Database (CDD) (https://ncbi.nlm.nih.gov/Structure/cdd/wrpsb.cgi), accessed on 2 February 2026.

### 4.6. Data Availability

The raw reads generated by HTS were deposited in NCBI SRA under accession numbers SRR32918043−SRR32918045, SRR39295637−SRR39295641, and SRR39299527. The sequences of virus genomes were deposited in NCBI GenBank under accession numbers PZ463385, PZ470505−PZ470529, PZ496784−PZ496801, and PZ507144.

## Figures and Tables

**Table 1:** Viruses found on symptomatic plants from phlox (*Phlox paniculata*) cultivar collections.

№ ^a^	Cultivar	Sample	Virus Detected ^b^	Genome Segment Sequenced ^c^	GenBank Accession Number	Reference
1	Mirage	Px15	AMV TSV BRSV BRSV satRNA	Complete genome Complete genome Complete genome Complete genome	PZ496784, PZ496785, PZ496786 PV524995, PV524996, PV524997 PV565078, PV565079 PV541178	This study [19] [19] [19]
2	Fuji	Px24	ArMV ArMV satRNA SpiMV3	Complete genome Complete genome Complete genome	PZ470521, PZ470522 PZ470510 PQ374234	This study This study [13]
3	Regina	Px31	AMV TSV BRSV	Complete genome Complete genome Complete genome	PZ496787, PZ496788, PZ496789 PV524998, PV52499, PV525000 PV565080, PV565081	This study [19] [19]
4	Elisabeth	Px32	TSV BRSV SpiMV3	RNA3 RNA1, RNA2 NIb-CP-3′UTR	PZ470508 PZ470519, PZ470520 PQ464120	This study This study [13]
5	Le Mahdi	Px35	TSV	ns		This study
6	Fliegelleutenant Bolke	Px50	TRV	RNA1	PZ496801	This study
7	Opal	Px51	AMV TSV	CP ns	PZ496790	This study This study
8	Dymchatyy Korall	Px52	AMV TSV BRSV BRSV satRNA SpiMV3	CP ns ns ns NIb-CP-3′UTR	PZ496791 PQ464121	This study This study This study This study [13]
9	Vladimir	Px53	AMV TSV CMV	ns ns CP	PZ470526	This study This study This study
10	Akkurat	Px54	AMV TSV CMV	ns ns CP	PZ470527	This study This study This study
11	Seyanets	Px55	AMV TSV SpiMV3	CP ns NIb-CP-3′UTR	PZ496792 PQ464122	This study This study [13]
12	Gegery	Px56	AMV TSV BRSV SpiMV3	ns ns ns NIb-CP-3′UTR	PQ464123	This study This study This study [13]
13	Festival’nyy	Px57	AMV TSV SpiMV3	ns ns NIb-CP-3′UTR	PQ464116	This study This study [13]
14	Vrubel	Px58	AMV TSV SpiMV3	RNA1, RNA2, RNA3 Complete genome P3…CP-3′UTR	PZ496794, PZ496795, PZ496796 PZ470505, PZ470506, PZ470507 PZ470509	This study This study This study
15	Moskovskie Zori	Px59	AMV TSV BRSV BRSV satRNA	CP ns ns ns	PZ496793	This study This study This study This study
16	Nikolas Flamel	Px60	AMV TSV BRSV BRSV satRNA	ns ns ns ns		This study This study This study This study
17	David	Px61	AMV TSV BRSV	ns ns ns		This study This study This study
18	Princessa Danii	Px62	TSV BRSV BRSV satRNA	Complete genome Complete genome Complete genome	PZ470511, PZ470512, PZ470513 PZ470517, PZ470518 PZ463385	This study This study This study
19	Leonid Vigorov	Px63	AMV TSV	ns ns		This study This study
20	Balerina	Px64	AMV TSV SpiMV3	ns ns NIb-CP-3′UTR	PQ464118	This study This study [13]
21	Ethel Prichard	Px65	AMV TSV	ns ns		This study This study
22	Belyy piramidal’nyy	Px66	AMV TSV	ns ns		This study This study
23	Pamyati Yermolovoy	Px67	AMV TSV CMV SpiMV3	ns ns CP NIb-CP-3′UTR	PZ470528 PQ464119	This study This study This study [13]
24	Belosnezhka	Px68	AMV TSV CMV	RNA1, RNA2, RNA3 Complete genome Complete genome	PZ496797, PZ496798, PZ496799 PZ470514, PZ470515, PZ470516 PZ470523, PZ470524, PZ470525	This study This study This study
25	Uspekh	Px69	AMV TSV	ns ns		This study This study
26	Nikolai Shchors	Px70	AMV TSV CMV	ns ns CP	PZ470529	This study This study This study
27	Unknown	PxBG2	AMV TRV TSV BRSV BRSV satRNA	Not assembled Complete genome Complete genome Complete genome Complete genome	PZ496800, PZ507144 PV525001, PV525002, PV525003 PV565076, PV5655077 PV541159	This study This study [19] [19] [19]

^a^ orange-colored cells indicate samples analyzed using high-throughput sequencing; ^b^ using high-throughput sequencing and/or RT-PCR. AMV, alfalfa mosaic virus; ArMV, arabis mosaic virus; ArMV sat RNA, ArMV satellite RNA; BRSV, beet ringspot virus; BRSV satRNA, BRSV satellite RNA; CMV, cucumber mosaic virus; SpiMV3, spiranthes mosaic virus 3; TRV, tobacco rattle virus; TSV, tobacco streak virus; ^c^ not assembled, the virus was detected by RT-PCR, but its genome failed to assemble; NIb-CP-3′UTR, entire coat protein (CP) gene flanked with adjacent segments of the NIb gene and 3′ untranslated region; ns, not sequenced; CP, coat protein; P3…CP-3′UTR, partial genome sequence spanning the P3 to CP genes and 3′ untranslated region.

## Data Availability

Sequencing data have been deposited in NCBI Sequence Read Archive and GenBank, and their accession numbers are provided within the article.

## Supplementary Materials

The following supporting information can be downloaded at: https://www.mdpi.com/article/10.3390/plants15172550/s1, Figure S1: Agarose gel electrophoresis of amplicons obtained by RT-PCR assay with virus-specific primers; Figure S2: Maximum likelihood phylogenetic trees based on full-length sequences of cucumber mosaic genome RNA1, RNA2, and RNA3; Table S1: Results of high-throughput sequencing of phlox samples with virus disease symptoms; Table S2: Virus-specific primers used for RT-PCR detection of viruses in phlox plants.
