# Supplementary material for: Prevalence, Species Diversity, and Molecular Characterization of Viruses Infecting Phlox (Phlox paniculata) from Botanical Gardens in Russia
Source: Plants (Basel). 2026 Aug 22;15(17):2550. doi: 10.3390/plants15172550 (PMC13567020; doi:10.3390/plants15172550)
Supplement: Supplementary file 1 [file plants-15-02550-s001.zip › Fig. S1.pdf]

A. Alfalfa mosaic virus

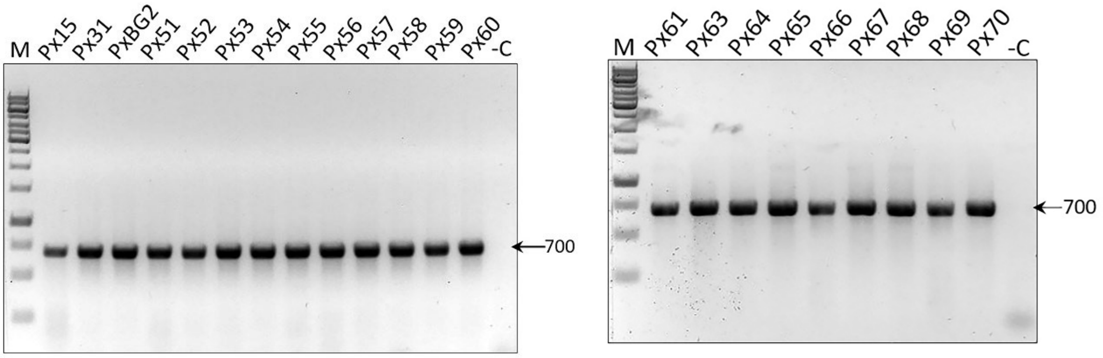

B. Tobacco streak virus

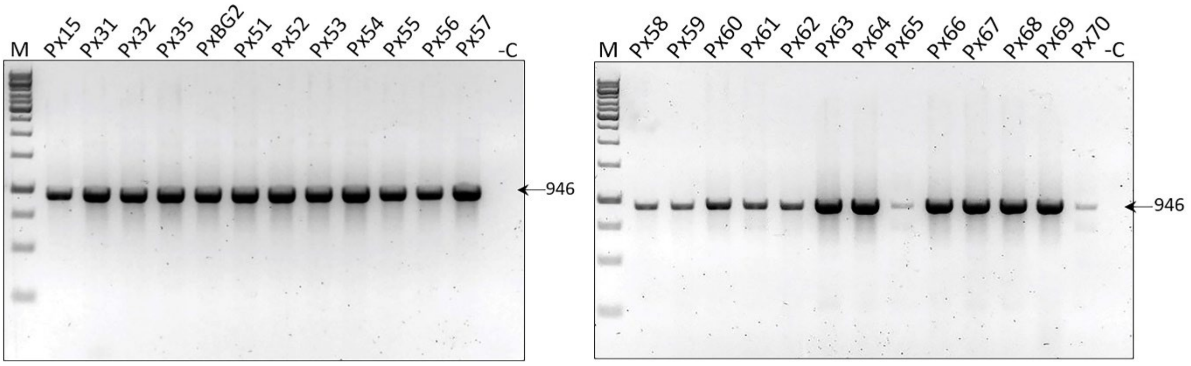

C. Beet ringspot virus

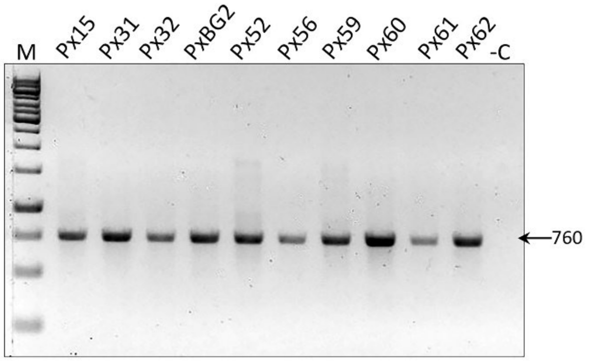

D. Beet ringspot virus satellite RNA

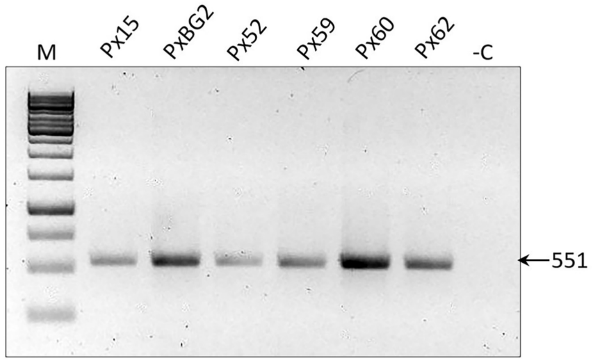

E. Cucumber mosaic virus

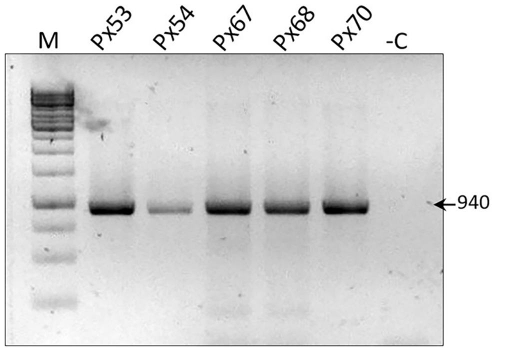

F. Tobacco rattle virus

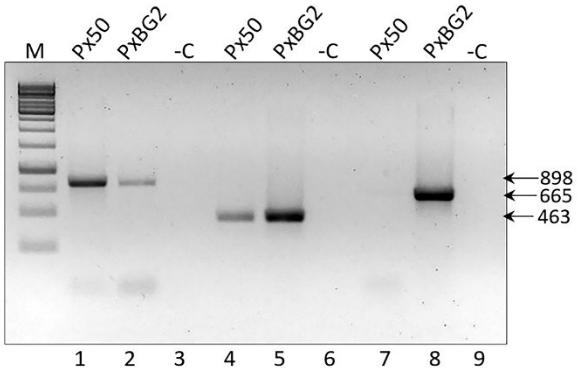

G. Arabis mosaic virus (ArMV) and ArMV satellite RNA

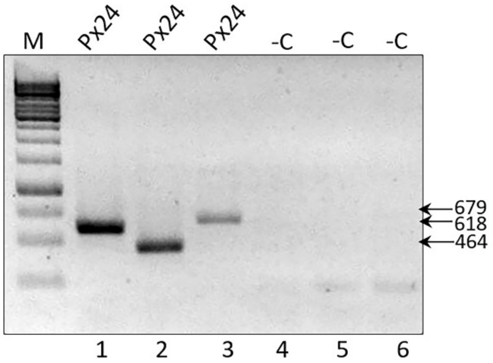

Figure S1. Agarose gel electrophoresis of amplicons obtained by RT-PCR assay with virus-specific primers to alfalfa mosaic (**A**), tobacco streak virus (**B**), beet ringspot virus (**C**), beet ringspot virus satellite RNA (**D**) and cucumber mosaic virus (**E**). **F**: PCR products obtained with primers targeting RNA-dependent RNA polymerase (898 bp, lanes 1-3), 16K (463 bp, lanes 4-6) and coat protein (665 bp, lanes 7-9) genes from tobacco rattle virus RNA1 and RNA2, respectively. **G**: PCR products obtained with primers specific to arabis mosaic virus (ArMV) RNA1 (618 bp, lanes 1, 4), RNA2 (464 bp, lanes 2, 5), and ArMV satellite RNA (679 bp, lanes 3, 6). -C — negative control (uninfected phlox). M — GeneRuler 1 kb DNA ladder (Thermo Scientific, Waltham, MA, USA). The arrows to the right of the figures indicate PCR products of corresponding sizes (bp).
