# Supplementary material for: Prevalence, Species Diversity, and Molecular Characterization of Viruses Infecting Phlox (Phlox paniculata) from Botanical Gardens in Russia
Source: Plants (Basel). 2026 Aug 22;15(17):2550. doi: 10.3390/plants15172550 (PMC13567020; doi:10.3390/plants15172550)
Supplement: Supplementary file 1 [file plants-15-02550-s001.zip › Fig. S2.pdf]

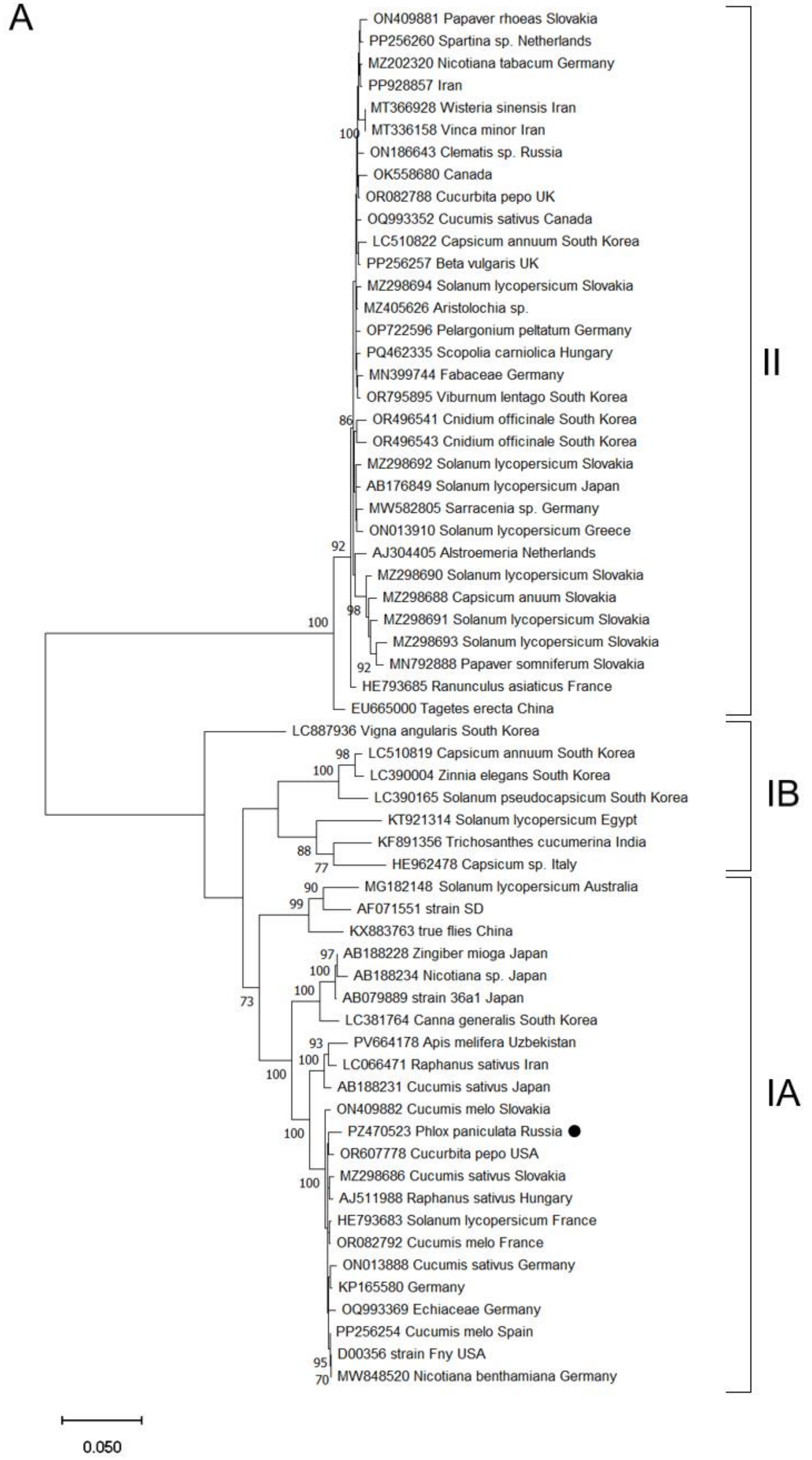
A
ON409881 Papaver rhoeas Slovakia
PP256260 Spartina sp. Netherlands
MZ202320 Nicotiana tabacum Germany
PP928857 Iran
MT366928 Wisteria sinensis Iran
MT336158 Vinca minor Iran
ON186643 Clematis sp. Russia
OK558680 Canada
OR082788 Cucurbita pepo UK
OQ993352 Cucumis sativus Canada
LC510822 Capsicum annuum South Korea
PP256257 Beta vulgaris UK
MZ298694 Solanum lycopersicum Slovakia
MZ405626 Aristolochia sp.
OP722596 Pelargonium peltatum Germany
PQ462335 Scopolia carniolica Hungary
MN399744 Fabaceae Germany
OR795895 Viburnum lentago South Korea
OR496541 Cnidium officinale South Korea
OR496543 Cnidium officinale South Korea
MZ298692 Solanum lycopersicum Slovakia
AB176849 Solanum lycopersicum Japan
MW582805 Sarracenia sp. Germany
ON013910 Solanum lycopersicum Greece
AJ304405 Alstroemeria Netherlands
MZ298690 Solanum lycopersicum Slovakia
MZ298688 Capsicum anuum Slovakia
MZ298691 Solanum lycopersicum Slovakia
MZ298693 Solanum lycopersicum Slovakia
MN792888 Papaver somniferum Slovakia
HE793685 Ranunculus asiaticus France
EU665000 Tagetes erecta China
LC887936 Vigna angularis South Korea
LC510819 Capsicum annuum South Korea
LC390004 Zinnia elegans South Korea
LC390165 Solanum pseudocapsicum South Korea
KT921314 Solanum lycopersicum Egypt
KF891356 Trichosanthes cucumerina India
HE962478 Capsicum sp. Italy
MG182148 Solanum lycopersicum Australia
AF071551 strain SD
KX883763 true flies China
AB188228 Zingiber mioga Japan
AB188234 Nicotiana sp. Japan
AB079889 strain 36a1 Japan
LC381764 Canna generalis South Korea
PV664178 Apis melifera Uzbekistan
LC066471 Raphanus sativus Iran
AB188231 Cucumis sativus Japan
ON409882 Cucumis melo Slovakia
PZ470523 Phlox paniculata Russia
OR607778 Cucurbita pepo USA
MZ298686 Cucumis sativus Slovakia
AJ511988 Raphanus sativus Hungary
HE793683 Solanum lycopersicum France
OR082792 Cucumis melo France
ON013888 Cucumis sativus Germany
KP165580 Germany
OQ993369 Echiaceae Germany
PP256254 Cucumis melo Spain
D00356 strain Fny USA
MW848520 Nicotiana benthamiana Germany
100
86
92
100
98
92
98
100
88
77
90
99
97
100
100
73
93
100
100
100
100
95
70
II
IB
IA
0.050

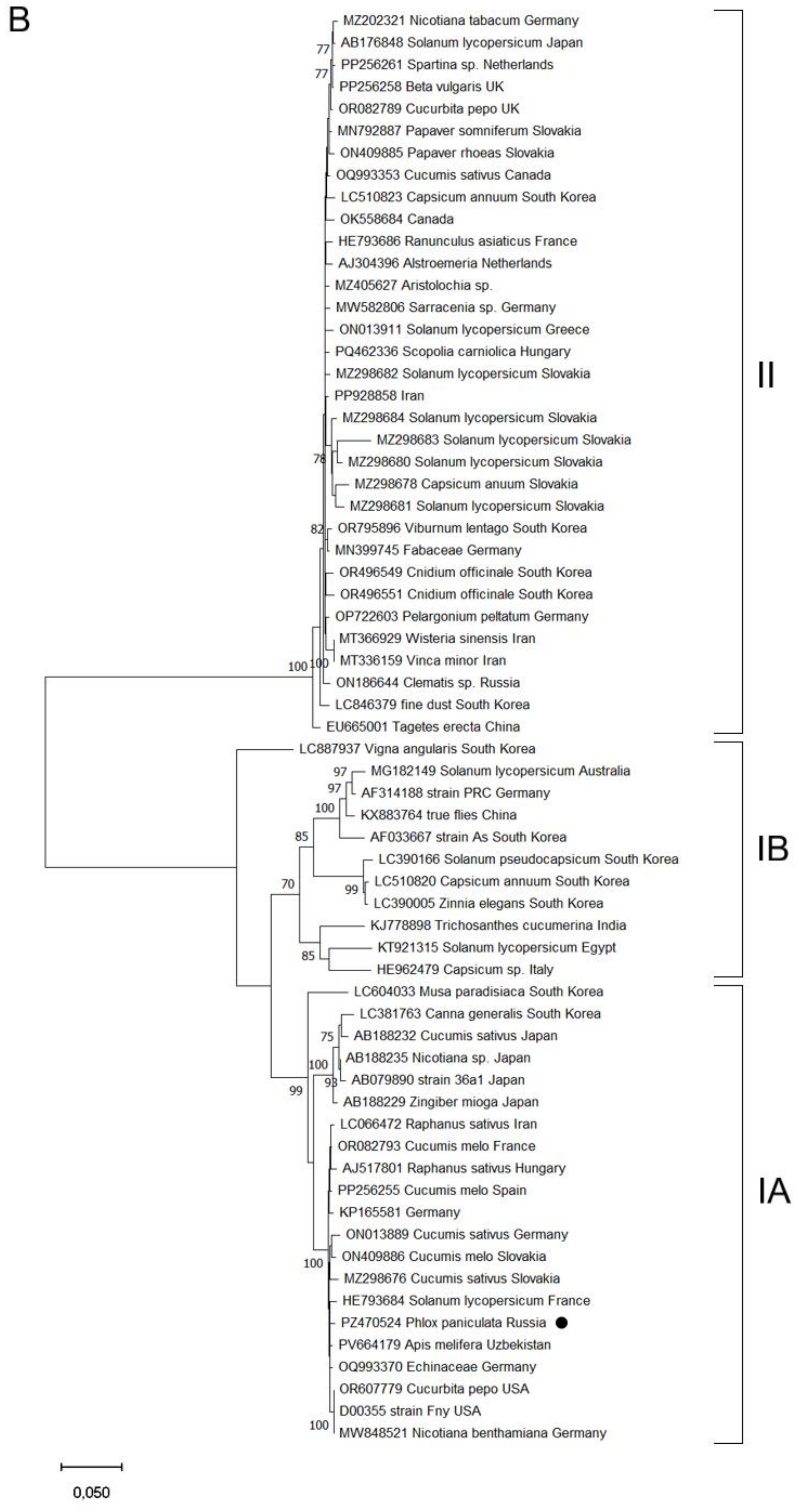
B
MZ202321 Nicotiana tabacum Germany
AB176848 Solanum lycopersicum Japan
PP256261 Spartina sp. Netherlands
PP256258 Beta vulgaris UK
OR082789 Cucurbita pepo UK
MN792887 Papaver somniferum Slovakia
ON409885 Papaver rhoeas Slovakia
OQ993353 Cucumis sativus Canada
LC510823 Capsicum annuum South Korea
OK558684 Canada
HE793686 Ranunculus asiaticus France
AJ304396 Alstroemeria Netherlands
MZ405627 Aristolochia sp.
MW582806 Sarracenia sp. Germany
ON013911 Solanum lycopersicum Greece
PQ462336 Scopolia carniolica Hungary
MZ298682 Solanum lycopersicum Slovakia
PP928858 Iran
MZ298684 Solanum lycopersicum Slovakia
MZ298683 Solanum lycopersicum Slovakia
MZ298680 Solanum lycopersicum Slovakia
MZ298678 Capsicum anuum Slovakia
MZ298681 Solanum lycopersicum Slovakia
OR795896 Viburnum lentago South Korea
MN399745 Fabaceae Germany
OR496549 Cnidium officinale South Korea
OR496551 Cnidium officinale South Korea
OP722603 Pelargonium peltatum Germany
MT366929 Wisteria sinensis Iran
MT336159 Vinca minor Iran
ON186644 Clematis sp. Russia
LC846379 fine dust South Korea
EU665001 Tagetes erecta China
LC887937 Vigna angularis South Korea
MG182149 Solanum lycopersicum Australia
AF314188 strain PRC Germany
KX883764 true flies China
AF033667 strain As South Korea
LC390166 Solanum pseudocapsicum South Korea
LC510820 Capsicum annuum South Korea
LC390005 Zinnia elegans South Korea
KJ778898 Trichosanthes cucumerina India
KT921315 Solanum lycopersicum Egypt
HE962479 Capsicum sp. Italy
LC604033 Musa paradisiaca South Korea
LC381763 Canna generalis South Korea
AB188232 Cucumis sativus Japan
AB188235 Nicotiana sp. Japan
AB079890 strain 36a1 Japan
AB188229 Zingiber mioga Japan
LC066472 Raphanus sativus Iran
OR082793 Cucumis melo France
AJ517801 Raphanus sativus Hungary
PP256255 Cucumis melo Spain
KP165581 Germany
ON013889 Cucumis sativus Germany
ON409886 Cucumis melo Slovakia
MZ298676 Cucumis sativus Slovakia
HE793684 Solanum lycopersicum France
PZ470524 Phlox paniculata Russia
PV664179 Apis melifera Uzbekistan
OQ993370 Echinaceae Germany
OR607779 Cucurbita pepo USA
D00355 strain Fny USA
MW848521 Nicotiana benthamiana Germany
II
IB
IA
0,050

C

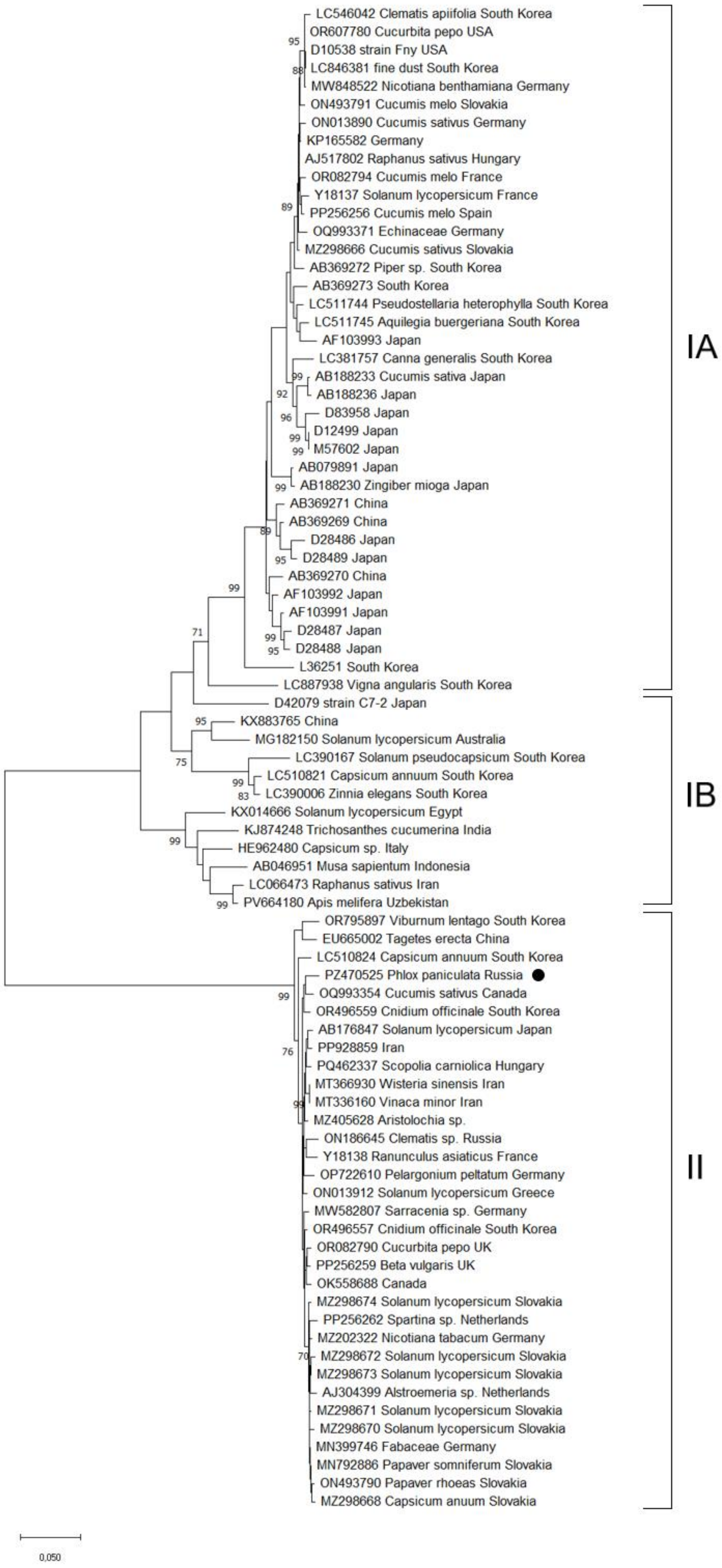
LC546042 Clematis apiifolia South Korea
OR607780 Cucurbita pepo USA
D10538 strain Fny USA
LC846381 fine dust South Korea
MW848522 Nicotiana benthamiana Germany
ON493791 Cucumis melo Slovakia
ON013890 Cucumis sativus Germany
KP165582 Germany
AJ517802 Raphanus sativus Hungary
OR082794 Cucumis melo France
Y18137 Solanum lycopersicum France
PP256256 Cucumis melo Spain
OQ993371 Echinaceae Germany
MZ298666 Cucumis sativus Slovakia
AB369272 Piper sp. South Korea
AB369273 South Korea
LC511744 Pseudostellaria heterophylla South Korea
LC511745 Aquilegia buergeriana South Korea
AF103993 Japan
LC381757 Canna generalis South Korea
AB188233 Cucumis sativa Japan
AB188236 Japan
D83958 Japan
D12499 Japan
M57602 Japan
AB079891 Japan
AB188230 Zingiber mioga Japan
AB369271 China
AB369269 China
D28486 Japan
D28489 Japan
AB369270 China
AF103992 Japan
AF103991 Japan
D28487 Japan
D28488 Japan
L36251 South Korea
LC887938 Vigna angularis South Korea
D42079 strain C7-2 Japan
KX883765 China
MG182150 Solanum lycopersicum Australia
LC390167 Solanum pseudocapsicum South Korea
LC510821 Capsicum annuum South Korea
LC390006 Zinnia elegans South Korea
KX014666 Solanum lycopersicum Egypt
KJ874248 Trichosanthes cucumerina India
HE962480 Capsicum sp. Italy
AB046951 Musa sapientum Indonesia
LC066473 Raphanus sativus Iran
PV664180 Apis melifera Uzbekistan
OR795897 Viburnum lentago South Korea
EU665002 Tagetes erecta China
LC510824 Capsicum annuum South Korea
PZ470525 Phlox paniculata Russia
OQ993354 Cucumis sativus Canada
OR496559 Cnidium officinale South Korea
AB176847 Solanum lycopersicum Japan
PP928859 Iran
PQ462337 Scopolia carniolica Hungary
MT366930 Wisteria sinensis Iran
MT336160 Vinaca minor Iran
MZ405628 Aristolochia sp.
ON186645 Clematis sp. Russia
Y18138 Ranunculus asiaticus France
OP722610 Pelargonium peltatum Germany
ON013912 Solanum lycopersicum Greece
MW582807 Sarracenia sp. Germany
OR496557 Cnidium officinale South Korea
OR082790 Cucurbita pepo UK
PP256259 Beta vulgaris UK
OK558688 Canada
MZ298674 Solanum lycopersicum Slovakia
PP256262 Spartina sp. Netherlands
MZ202322 Nicotiana tabacum Germany
MZ298672 Solanum lycopersicum Slovakia
MZ298673 Solanum lycopersicum Slovakia
AJ304399 Alstroemeria sp. Netherlands
MZ298671 Solanum lycopersicum Slovakia
MZ298670 Solanum lycopersicum Slovakia
MN399746 Fabaceae Germany
MN792886 Papaver somniferum Slovakia
ON493790 Papaver rhoeas Slovakia
MZ298668 Capsicum anuum Slovakia
IA
IB
II
0,050

Figure S2. Maximum likelihood phylogenetic trees based on full-length sequences of cucumber mosaic virus genome RNA1 (**A**), RNA2 (**B**), and RNA3 (**C**) using the GTR best-fit substitution model. The accession number, Latin name of the host plant, and geographical origin of the virus isolate are given at the end of the branches. Russian phlox isolate is marked with a black circle (•). Bootstrap values from 1000 replications (>70%) are shown next to the corresponding node. The scale bar indicates the number of nucleotide substitutions per site.
