# Supplementary material for: Prevalence, Species Diversity, and Molecular Characterization of Viruses Infecting Phlox (Phlox paniculata) from Botanical Gardens in Russia
Source: Plants (Basel). 2026 Aug 22;15(17):2550. doi: 10.3390/plants15172550 (PMC13567020; doi:10.3390/plants15172550)
Supplement: Supplementary file 1 [file plants-15-02550-s001.zip › Table S1.pdf]

Table S1. Results of high-throughput sequencing of phlox samples with symptoms of virus diseases

Sample	Accession Biosample[1]	Accession SRA[2]	Number of reads[3]		Number of assembled contigs	
			before filter	after filter	Total	Virus-related
Px24	SAMN61081410	SRR39295637	22.152208 M	21.659478 M	43,703	225
Px32	SAMN61081409	SRR39295638	99.422 K	95.672 K	325	55
Px68	SAMN61081408	SRR39295639	34.054312 M	33.714704 M	26,134	107
Px62	SAMN61081407	SRR39295640	48.385662 M	47.922546 M	35,754	93
Px58	SAMN61081406	SRR39295641	9.243680 M	9.151970 M	15,121	50
Px50	SAMN61081405	SRR39299527	48.552832 M	48.052282 M	60,943	85
Px15	SAMN47635716	SRR32918045	40.262202 M	39.594680 M	39,011	668
Px31	SAMN47635717	SRR32918044	35.663968 M	34.920082 M	45,360	645
PxBG2	SAMN47635718	SRR32918043	51.817462 M	50.223590 M	10,919	218

[1] BioSample — NCBI BioSample database accession number (https://www.ncbi.nlm.nih.gov/biosample/).
[2] SRA — Sequence Read Archive, an NCBI repository storing raw sequencing data (https://www.ncbi.nlm.nih.gov/sra/).
[3] M — million reads; K — thousand reads.
