# Supplementary material for: Prevalence, Species Diversity, and Molecular Characterization of Viruses Infecting Phlox (Phlox paniculata) from Botanical Gardens in Russia
Source: Plants (Basel). 2026 Aug 22;15(17):2550. doi: 10.3390/plants15172550 (PMC13567020; doi:10.3390/plants15172550)
Supplement: Supplementary file 1 [file plants-15-02550-s001.zip › Table S2.pdf]

Table S2. Virus-specific primers used for RT-PCR detection of viruses in phlox plants

№	Virus[a]	Target RNA/gene[b]	Primer	Sequence, 5'..3'	PCR product, bp	Cycling conditions (denaturation/annealing/ elongation), 35 cycles	Reference
1	ArMV	RNA1/RdRp	Ar1-F<br>Ar1-R	ATGGGTCAGATATGCTGCAGA<br>CATACAATGCAAGGGTGTCCT	618	94°C 30 s/50°C 30 s/ 72°C 40 s	This study
		RNA2/CP	Ar2-F<br>Ar2-R	GAGAAACAGCTAAGATGCCAGT<br>TAGCAAGCCATCGGCTGTACA	464	94°C 30 s/52°C 30 s/ 72°C 35 s	
2	ArMV satRNA	satRNA/ORF1	stArMV-F<br>stArMV-R	TAAGCAGCTCACTCGCATGCTT<br>ACTAGCCATGTCATCGGAGGTT	679	94°C 30 s/54°C 30 s/ 72°C 45 s	This study
3	TRV	RNA1/RdRp	Tobra-F3<br>Tobra-R2	GGTGGKCAATGGTCTTWTTGG<br>GTCAGCTGYTGATCAGATAACC	898	94°C 30 s/58°C 30 s/ 72°C 1 min	[33]
		RNA1/P1b	TRV-A<br>TRV-B	CAGTCTATACACAGAAACAGA<br>GACGTGTGTACTCAAGGGTT	463	94°C 30 s/58°C 30 s/ 72°C 35 s	[34]
		RNA2/CP	pTRV-CP-F<br>pTRV-CP-R	CTGGTTACTGAATCACTTACGCT<br>CGACATCAAGGATTAGGACGCA	665	94°C 30 s/50°C 30 s/ 72°C 40 s	This study
4	CMV	RNA3/CP	CPTALL-5<br>CPTALL-3	YASYTTTDRGGTTCAATTCC<br>GACTGACCATTTTAGCCG	940	94°C 30 s/46°C 30 s/ 72°C 1 min	[31]
5	AMV	RNA3/CP	AMVcoat-F<br>AMVcoat-R	GTGGTGGGAAAGCTGGTAAA<br>CACCCAGTGGAGGTCAGCATT	700	94°C 30 s/48°C 30 s/ 72°C 45 s	[8]
6	TSV	RNA3/CP	PxTSV-CP-F<br>PxTSV-CP-R	AGTCGCTTCTCGGACTTACCT<br>GGAAATCGTCCGATTCGGAAT	946	94°C 30 s/50°C 30 s/ 72°C 1 min	[19]
7	BRSV	RNA1/RdRp	PxBRSV-F<br>PxBRSV-R	TGAACTTAAGAGCCAAATTGAATA<br>GACAAGGCAAGACATGTCTATT	760	94°C 30 s/50°C 30 s/ 72°C 50 s	[19]
8	BRSV satRNA	satRNA/p48	stBRSV-F<br>stBRSV-R	AGGGTATGTACGCCAGTTCCT<br>GCTCTGGAGAAAAGGTATACAAT	551	94°C 30 s/50°C 30 s/ 72°C 45 s	[19]
9	SpiMV3	RNA/CP	Forward<br>Reverse	GCTTATATCAAGATGAGAAATAGC<br>GTGTAGGTAGAGAGACAGAAAC	370	94°C 30 s/52°C 30 s/ 72°C 30 s	[30]

[a]ArMV - arabis mosaic virus; ArMV satRNA – ArMV satellite RNA; TRV - tobacco rattle virus; CMV - cucumber mosaic virus; AMV - alfalfa mosaic virus; TSV - tobacco streak virus; BRSV - beet ringspot virus; BRSV satRNA – BRSV satellite RNA, SpiMV3 - spiranthes mosaic virus 3.

[b]RdRp – RNA-dependent RNA polymerase, CP – coat protein, P1b – 16K silencing suppressor protein, p48 - non-structural protein essential for satRNA replication.
